# The prevalence of headache disorders in children and adolescents in Lithuania: a schools-based study

**DOI:** 10.1186/s10194-020-01146-x

**Published:** 2020-06-10

**Authors:** Diana Genc, Nerija Vaičienė-Magistris, Apolinaras Zaborskis, Tayyar Şaşmaz, Aylin Yeniocak Tunç, Derya Uluduz, Timothy J. Steiner

**Affiliations:** 1 Department of Neurology, Faculty of Medicine, Lithuanian University of Health Sciences, Kaunas, Lithuania; 2 Department of Preventive Medicine and Health Research Institute, Faculty of Public Health, Lithuanian University of Health Sciences, Kaunas, Lithuania; 3 Department of Public Health, Mersin University School of Medicine, Mersin, Turkey; 4 Neurology Department, Cerrahpaşa School of Medicine, Istanbul University, Istanbul, Turkey; 5 Department of Neuromedicine and Movement Science, Norwegian University of Science and Technology, Edvard Griegs gate, Trondheim, Norway; 6 Division of Brain Sciences, Imperial College London, London, UK

**Keywords:** **C**hild and adolescent headache, Migraine, Tension-type headache, Medication-overuse headache, Undifferentiated headache, Epidemiology, Prevalence, Schools-based study, Lithuania, Global campaign against headache

## Abstract

**Background:**

While the Global Burden of Disease (GBD) study reports headache disorders as the second-highest cause of disability worldwide, the headache data in GBD come very largely from adults. This national study in Lithuania was part of a global schools-based programme within the Global Campaign against Headache contributing data from children (7–11 years) and adolescents (12–17 years).

**Methods:**

The methods followed the generic protocol for the global study. The basic study design was a cross-sectional survey. Self-completed structured questionnaires were administered, within classes, in 24 schools selected from seven regions of Lithuania to be nationally representative. Headache diagnostic questions were based on ICHD-3 beta criteria but for the inclusion of undifferentiated headache (UdH).

**Results:**

Of 3714 potential participants, 2505 (children 1382 [55.2%], adolescents 1123 [44.8%]; males 1169 [46.7%], females 1336 [53.3%]) completed the questionnaire. Adolescents and males were therefore relatively under-represented, with non-participation (32.6%) due in most cases to lack of parental consent. Observed lifetime prevalence of headache was 92.2%. Gender- and age-adjusted 1-year prevalence was 76.6% (migraine: 21.4%; tension-type headache [TTH]: 25.6%; UdH: 24.0%; all headache on ≥15 days/month: 3.9%; probable medication-overuse headache: 0.8%). All headache types except UdH were more prevalent among females than males, and among adolescents than children. UdH showed a complex relationship with age, but represented 38.0% of all reported headache in children, 27.4% in adolescents. Headache yesterday (HY) was reported by 17.5%, almost double the 9.8% predicted from prevalence and headache frequency to have headache on any day. The reason was unclear.

**Conclusions:**

Findings were not very different from those reported in Turkey and Austria, but with more TTH. Headache has, therefore, again been shown to be common in children and adolescents, and UdH confirmed as a headache type that must be recognised and included in accounts of headache in these age groups.

## Introduction

While multiple iterations of the Global Burden of Disease (GBD) study have shown headache disorders to be a major cause of public ill health worldwide [1–6], the data for headache in GBD have, so far, come very largely from adults. The prevalence and burden of headache disorders in children and adolescents are not well characterised and in many parts of the world are unmeasured.

A global study of child and adolescent headache is consequently underway: it is an essential part of the endeavour by the Global Campaign against Headache, under the direction of *Lifting The Burden* (LTB) [7–10], to measure the scale and scope of headache-attributed burden worldwide [7–9]. The intention, in this schools-based programme, is to cluster-sample the world, gathering data according to a standardised protocol [11] from at least 20 countries spread through the world’s six major regions. The protocol was piloted in Turkey and Austria [11–13].

The programme focuses on the headache disorders with public-health importance not only in children and adolescents but also in adults: migraine, tension-type headache (TTH) and the group of headache disorders characterised by headache occurring on ≥15 days per month, including medication-overuse headache (MOH). These disorders in adults have very commonly had their onset in pre-adult years. However, the studies from Turkey and Austria showed a high prevalence of undifferentiated headache (UdH), not diagnosable as migraine or TTH [12, 13], which is therefore included in studies within this programme. By definition, this type of headache is mild and of short duration (< 1 h) [12].

This national study in Lithuania, of children aged 7–11 years and adolescents aged 12–17 years, was a part of the global schools-based programme. Because institutional schooling is mandatory for 10 years in Lithuania, from ages 6.5 or 7.5 years depending on month of birth, and about 90% of pupils continue for another 2 years, schools-based sampling was methodologically valid for these age groups [14]. The aims of the study were to estimate the prevalences of these headache disorders in these age groups, reported here, and their attributed burden, to be reported later. The purposes were to inform not only GBD but also local health policy.

## Methods

The basic study design was a cross-sectional survey conducted by self-completed structured questionnaire administered in schools selected to be nationally representative. The methods have been published earlier [15], as has the generic protocol [11].

### Ethics and approvals

The study was approved by Kaunas Regional Committee of Bioethics (BE-2-7, 26-01-2016), and authorised, as required, by Regional Education Authorities.

School managers and teachers at the selected schools agreed to participation. Consent of each participating child or adolescent was obtained prior to recruitment, along with written parental consent. For these purposes, information sheets were prepared for potential participants and their parents, describing the nature and purposes of the survey. These were distributed to pupils in the participating schools 1 week before the study start. On the day of the study, pupils who were willing, and possessed a consent form signed by a parent, were invited to complete the questionnaire.

Data were collected anonymously. Data protection legislation was complied with.

### Sampling and recruitment

The survey was conducted in Spring, 2016.

**Fig. 1:**
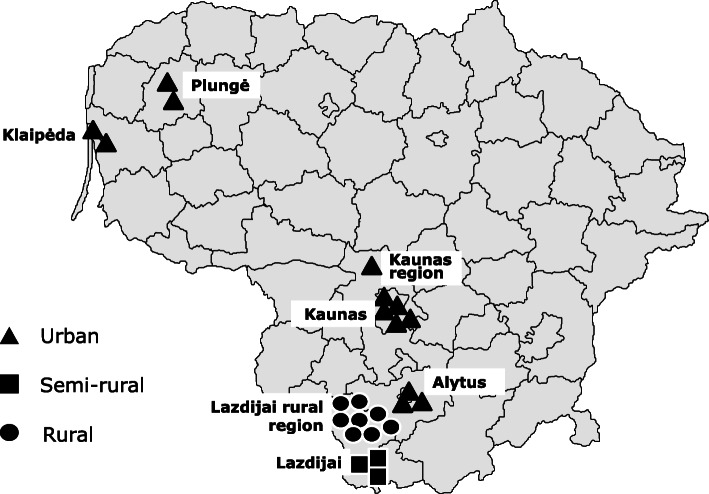
Location of 24 selected schools within seven regions of Lithuania

We randomly selected 24 schools located in seven regions of Lithuania (Fig. 1), the latter purposively chosen to represent the country’s limited geographical and socioeconomic diversities. All classes in each school across the age ranges 7–11 years and/or 12–17 years, and all pupils in each such class, except those for whom parental consent had not been given, were invited to participate. Pupils who refused on their own account to take part for any reason were excluded, along with those absent from school on the day. All but the last group were counted as non-participants.

### Numbers

Published recommendations on sample size calculation for headache *prevalence* [14] suggest limited gain from samples greater than *N* = 2000. For *burden*, larger numbers may be more informative, but there is no good basis for power calculation, given the multiple components of burden [14]. Our aim here was for 200 evaluable participants of each age 7–17 years, drawn approximately equally from the participating schools (expected totals of 1000 children and 1200 adolescents).

### Survey instrument

We employed the child and adolescent versions of LTB’s Headache-Attributed Restriction, Disability, Social Handicap and Impaired Participation (HARDSHIP) structured questionnaire [11], translated into Lithuanian language according to LTB’s translation protocol for hybrid documents [16]. These modular instruments, designed for application by lay interviewers, incorporated demographic enquiry, headache screening and diagnostic questions based on ICHD-3 beta criteria [17] and enquiries into components of headache-attributed burden. The timeframe of enquiry was the preceding 4 weeks (28 days), except for the module asking specifically about headache yesterday (HY).

These questionnaires were administered to the pupils in class, who completed them anonymously under the supervision of an investigator, a previously instructed school public-health specialist and/or a teacher. After an initial introduction, adolescents, and children competent in reading, completed their questionnaires independently, without further assistance. Children aged 7 or 8 years, and a few older children with learning difficulties, were assisted as necessary.

Additional questionnaires addressed to teachers enquired into relevant school variables.

### Diagnoses

Diagnoses were made algorithmically according to HARDSHIP methodology [11, 18]. Headache on ≥15 days/month (*ie*, reported on ≥14 days in the preceding 4 weeks) was first identified, and categorised according to reported medication use into probable MOH (pMOH: use on ≥14 days/4 weeks) or “other headache on ≥15 days/month” (use on 0–13 days/4 weeks). To all other reported headaches, the algorithm first applied criteria for UdH (mild intensity and usual duration < 1 h [12]) and then the ICHD-3 beta criteria [17], with one important modification, for definite migraine, definite TTH, probable migraine and probable TTH in this strict order [18]. The modification, to accommodate the definition of UdH, was to raise the lower duration limit of TTH from 30 min to 1 h [12]. Remaining cases, if any, were unclassified.

### Data management and entry

Completed questionnaires were removed to the Department of Preventive Medicine and Health Research Institute at Lithuanian University of Health Sciences in Kaunas, and held securely during data entry into an electronic database. As a quality-control measure, we performed independent double data-entry with reconciliation of discrepancies by comparison with source data.

### Analysis

Analyses were performed at University of Mersin.

We categorised schools by locality (urban, semi-rural, rural) and by estimated proportions of pupils coming from low-income homes (< 0.25, 0.25–0.5, 0.5–0.75 [categories referred to for simplicity as “high-income”, “middle-income” and “low-income”]). We used descriptive statistics to present means and standard deviations (SDs) of continuous variables and proportions (%) with 95% confidence intervals (CIs) of categorical data. We used chi-squared or one-sample proportion tests to evaluate differences between groups. We estimated prevalences of each headache type as proportions (%) with 95% CIs, and adjusted the observed values for gender and age using official population statistics for Lithuania [19]. In these analyses, definite and probable migraine were combined, as were definite and probable TTH [14, 18]. To show associations with demographic variables, we first used bivariate analysis with odds ratios (ORs), then multivariate analysis with adjusted ORs (AORs), entering gender, age group, school locality and income category into the multivariate model. Mean headache frequencies (days/month) were calculated to support estimates of predicted headache yesterday.

We considered *p* < 0.05 to be significant.

## Results

According to class lists, the sampled population consisted of 4019 children and adolescents (male 2016 [50.2%], female 2003 [49.8%]), but 305 (7.6%) were absent on the day. Of the 3714 potential participants, 897 (24.2%) did not or could not provide parental consent and another 312 (8.4%) did not themselves consent, with more males (55.9%) than females (44.1%) among these. The non-participation proportion was 32.6%. Among actual participants (*N* = 2505; male 1169 [46.7%], female 1336 [53.3%]), males were under-represented (46.7% against 50.2% expected; females 53.3% against 49.8% expected; one sample proportion test: *p* < 0.001 [2-tailed]).

There were 1382 children (55.2%) and 1123 adolescents (44.8%), with overall mean age of 11.5 ± 3.2 years (median 11). Since we intended to have similar numbers of each age group, children were relatively over-sampled. School variables are shown in Table 1. Most participants (66.3%) attended urban schools. A substantial minority (20.3%) attended schools where > 50% of pupils were from low-income homes, these also being the rural schools (Table 1).

**Table 1:** School variables (from teachers’ questionnaires), and numbers of participant pupils affected (*N* = 2505)

Variable	Schools	Pupils	
	n	n	%
School locality			
urban	13	1660	66.3
semi-rural	3	336	13.4
rural	8	509	20.3
Income category according to estimated proportion of pupils from low-income homes			
< 0.25 (“high-income” school)	13	1828	73.0
0.25–0.5 (“middle-income” school)	3	168	6.7
0.5–0.75 (“low-income” school)	8	509	20.3

### Headache

Headache ever was reported by 2309 participants (observed lifetime prevalence = 92.2%). Headache in the preceding year was reported by 1858 (observed 1-year prevalence = 74.2%; gender- and age-adjusted: 76.6%). Observed and adjusted prevalences of each headache type are shown in Table 2.

**Table 2:** Crude (observed) prevalences of all headache and each headache type, overall and according to demographic variables, and gender- and age-adjusted prevalences (*N* = 2505)

	All headache (*n* = 1858)	Migraine (*n* = 519)	TTH (*n* = 605)	pMOH (*n* = 18)	Other headache on ≥ 15 d/m (*n* = 73)	UdH (*n* = 601)
**Observed prevalences** (% [95% CI])						
Overall	74.2 [72.5–75.9]	20.7 [19.1–22.3]	24.2 [22.5–25.9]	0.7 [0.4–1.0]	2.9 [2.2–3.6]	24.0 [22.3–25.7]
Gender						
male (*n* = 1169)	67.8 [65.1–70.5]	18.6 [16.4–20.8]	22.3 [19.9–24.7]	0.2 [0.0–0.5]	1.5 [0.8–2.2]	24.0 [21.7–26.3]
female (*n* = 1336)	79.7 [77.5–81.9]	22.5 [20.3–24.7]	25.8 [23.5–28.1]	1.2 [0.6–1.8]	4.2 [3.1–5.3]	24.0 [21.6–26.5]
Age group (years)						
7–11 (*n* = 1382)	62.7 [60.2–65.2]	17.9 [15.9–19.9]	17.5 [15.5–19.5]	0.3 [0.0–0.6]	1.3 [0.7–1.9]	23.8 [21.6–26.1]
12–17 (*n* = 1123)	88.3 [86.4–90.2]	24.2 [21.7–26.7]	32.3 [29.6–35.0]	1.3 [0.6–2.0]	4.9 [3.6–6.2]	24.2 [21.7–26.7]
School income category*						
high (*n* = 1828)	75.6 [73.6–77.6]	21.8 [19.9–23.7]	25.7 [23.7–27.7]	0.7 [0.3–1.1]	3.1 [2.3–3.9]	22.5 [20.6–24.4]
middle (*n* = 168)	61.9 [54.6–69.2]	20.8 [14.7–26.9]	16.1 [10.5–21.7]	0.6 [0.0–1.8]	1.2 [0.0–2.8]	22.0 [15.7–28.3]
low (*n* = 509)	73.1 [69.2–77.0]	16.9 [13.6–20.2]	21.4 [17.8–25.0]	0.8 [0.0–1.6]	2.9 [1.4–4.4]	29.9 [25.9–33.9]
School locality						
urban (*n* = 1660)	73.3 [71.2–75.4]	21.6 [19.6–23.6]	24.8 [22.7–26.9]	0.7 [0.3–1.1]	2.5 [1.7–3.3]	22.0 [20.0–24.0]
semi-rural (*n* = 336)	79.5 [75.2–83.8]	22.3 [17.8–26.8]	25.3 [20.7–29.9]	0.9 [0.0–1.9]	4.8 [2.5–7.1]	24.7 [20.1–29.3]
rural (*n* = 509)	73.1 [69.2–77.0]	16.9 [13.6–20.2]	21.4 [17.8–25.0]	0.8 [0.0–1.6]	2.9 [1.4–4.4]	29.9 [25.9–33.9]
**Gender and age-adjusted prevalences** (% [95% CI])						
Overall	76.6 [74.9–78.3]	21.4 [19.8–23.0]	25.6 [23.9–27.3]	0.8 [0.5–1.2]	3.1 [2.4–3.8]	24.0 [22.3–25.7]

*TTH* tension-type headache, *pMOH* probable medication-overuse headache, *d/m* days/month, *UdH* undifferentiated headache, *CI* confidence interval; * see text or Table 1 for explanation. Tables 3 and 4 provide odds ratios and adjusted odds ratios for these comparisons

TTH (25.6)% and UdH (24.0%) were the most common headache types after adjustment, with migraine not very far behind (21.4%) (Table 2). Headache on ≥15 days/month affected almost one participant in 25 (3.9%), who reported headache on a mean of 18.7 days/4 weeks. These included 0.8% with pMOH, who reported headache and medication use on 19.7 and 18.2 days/4 weeks respectively. There were 42 headaches cases (1.7%) remaining unclassified.

### Demographic associations

These are illustrated in Tables 2, 3, 4. All headache types except UdH were more prevalent among females than males, and among adolescents than children. UdH showed a complex relationship with age. In absolute terms, its prevalence increased from 19.3% at ages 7–8 years to a maximum of 30.7% at ages 11–12, then declined to a minimum of 18.1% at 17 years. This was against a background of increasing prevalence of all other headache types, so that UdH represented 38.0% of all reported headache in children, but only 27.4% in adolescents. Headache on ≥15 days/month (pMOH: OR = 5.0; other: OR = 3.5 [Table 3]) was much more common among adolescents.

**Table 3:** Bivariate logistic regression analyses of headache type versus demographic variables (*N* = 2505)

Variable	Migraine	TTH	pMOH	Other headache on ≥ 15 d/m	UdH
	Odds ratio [95% CI]				
Gender					
male (*n* = 1169)	reference	reference	reference	reference	reference
female (*n* = 1336)	1.2 [1.0–1.5]	**1.2** [1.01–1.5]^**1**^	**7.1** [1.6–30.8]^**2**^	**3.0** [1.7–5.1]^**4**^	1.0 [0.8–1.2]
Age group (years)					
7–11 (*n* = 1382)	reference	reference	reference	reference	reference
12–17 (*n* = 1123)	**1.5** [1.2–1.8]^**4**^	**2.2** [1.9–2.7]^**4**^	**4.3** [1.4–13.2]^**2**^	**3.9** [2.3–6.7]^**4**^	1.0 [0.85–1.2]
School income category*					
high (*n* = 1828)	reference	reference	reference	reference	reference
middle or low (*n* = 677)	**0.8** [0.6–0.98]^**1**^	**0.7** [0.6–0.9]^**2**^	1.0 [0.4–2.9]	0.8 [0.5–1.4]	**1.3** [1.1–1.6]^**2**^
School locality					
urban (*n* = 1660)	reference	reference	reference	reference	reference
semi-rural (*n* = 336)	1.0 [0.8–1.4]	1.0 [0.8–1.3]	1.4 [0.4–4.9]	**1.9** [1.1–3.5]^**1**^	1.2 [0.9–1.5]
rural (*n* = 509)	**0.7** [0.6–0.96]^**1**^	0.8 [0.7–1.05]	1.2 [0.4–3.7]	1.2 [0.6–2.1]	**1.5** [1.2–1.9]^**3**^

*TTH* tension-type headache, *pMOH* probable medication-overuse headache, *d/m* days/month, *UdH* undifferentiated headache, *CI* confidence interval; * see text or Table 1 for explanation; significant values are emboldened: ^1^*p* < 0.05; ^2^*p* < 0.01; ^3^*p* < 0.001; ^4^*p* ≤ 0.0001

**Table 4:** Multivariate logistic regression analyses of headache type versus demographic variables (*N* = 2505)

Variable	Migraine	TTH	pMOH	Other headache on ≥ 15 d/m	UdH
	Adjusted odds ratio [95% CI]*				
Gender					
male (*n* = 1169)	reference	reference	reference	reference	reference
female (*n* = 1336)	**1.25** [1.03–1.5]^**1**^	1.2 [0.97–1.4]	**6.8** [1.6–28.9]^**1**^	**2.8** [1.6–4.9]^**1**^	0.99 [0.8–1.2]
Age group (years)					
7–11 (*n* = 1382)	reference	reference	reference	reference	reference
12–17 (*n* = 1123)	**1.5** [1.2–1.9]^**2**^	**2.25** [1.85–2.75]^**2**^	**5.0** [1.4–17.7]^**1**^	**3.5** [2.0–6.2]^**2**^	0.99 [0.8–1.2]
School income category*					
high (*n* = 1828)	0.8 [0.5–1.2]	1.1 [0.7–1.75]	0.3 [0.03–3.6]	1.3 [0.3–6.1]	1.1 [0.7–1.7]
middle or low (*n* = 677)	reference	reference	reference	reference	reference
School locality					
urban (*n* = 1660)	reference	reference	reference	reference	reference
semi-rural (*n* = 336)	0.9 [0.7–1.2]	0.9 [0.65–1.2]	0.9 [0.2–3.3]	1.5 [0.8–2.8]	1.2 [0.9–1.6]
rural (*n* = 509)	**0.6** [0.36–0.96]^**1**^	0.9 [0.5–1.4]	0.4 [0.03–5.0]	1.4 [0.3–7.3]	**1.7** [1.06–2.7]^**1**^

*TTH* tension-type headache, *pMOH* probable medication-overuse headache, *d/m* days/month, *UdH* undifferentiated headache, *CI* confidence interval; * see text or Table 1 for explanation; significant values are emboldened: ^1^*p* < 0.05; ^2^*p* < 0.001

Associations with income category apparent on bivariate analysis (Table 3) lost significance in multivariate analysis, with location factored in (Table 4). Migraine was less prevalent, and UdH more prevalent, in rural schools than others (Tables 2, 3, 4).

### Headache yesterday (HY)

HY was reported by 438 participants (17.5% of the total sample; 23.6% of those with any headache) (Table 5).

**Table 5:** Proportions reporting headache yesterday, and predicted proportions^a^, overall and by headache type (*N* = 2505)

Headache type	Headache yesterday		
	Reported proportion n (%)	Predicted proportion	
		Mean reported headache frequency (F) (days/4 weeks)	Predicted headache yesterday^**a**^ (%)
**Any headache** (*n* = 1858)	438 (23.6)	3.7 ± 4.5	13.2
**Migraine** (*n* = 519)	160 (30.8)	3.9 ± 3.3	13.9
**TTH** (*n* = 605)	127 (21.0)	3.2 ± 3.0	11.4
**pMOH** (*n* = 18)	13 (72.2)	19.7 ± 4.1	70.4
**Other headache on ≥ 15 d/m** (*n* = 73)	58 (79.5)	18.4 ± 4.3	65.7
**UdH** (*n* = 601)	68 (11.3)	1.9 ± 2.2	6.6

^a^calculated as F/28; *TTH* tension-type headache, *pMOH* probable medication-overuse headache, *d/m* days/month, *UdH* undifferentiated headache

Mean headache frequency in the 1858 reporting any headache was 3.7 ± 4.5 days/4 weeks. Probability of HY among these, calculated as proportion of days affected, was 13.2% (3.7/28). Since observed prevalence of all headache = 74.2% (Table 2), 9.8% of all participants (74.2*13.2%) would be expected to have headache on any day (predicted HY). In fact, participants with all episodic headache types reported substantially more HY than predicted, notably those with migraine (by a factor of 2.2) (Table 5). Headache on ≥15 days/month, particularly pMOH, was reported yesterday only slightly more than predicted. These disorders were, of course, the greatest contributors proportionately to HY (by definition, > 50% would be expected), but their low prevalence limited their overall impact. Females reported HY more than males (OR: 2.0 [1.6–2.5]; *p* < 0.001), but adolescents no more than children (OR; 1.1 [0.9–1.3]; *p* = 0.5727).

## Discussion

This was the second national study of child and adolescent headache to be completed within the Global Campaign programme of such studies. In summary, it found headache to be very common in these age groups in Lithuania, with over three quarters (76.6%) affected. TTH (25.6%) and UdH (24.0%) accounted for most, but migraine was also prevalent (21.4%). Headache on ≥15 days/month was far from rare (3.9%), although pMOH was found in only 0.8%. HY was reported by more than one in six participants (17.5%). Associations with age and gender were much as expected, although we say more below about the relationship between UdH and age. Migraine was less prevalent, and UdH more prevalent, suggesting a lower rate of headache differentiation, in rural schools than others. We attach no importance to this. There were no clear associations with income category.

Lithuania and Turkey (venue for the first study) are both in the European Region, but not close geographically, ethnically or culturally. Comparisons between these are of particular interest because the studies in each used the same methodology and questionnaires. The Turkish study (*N* = 7088) has not yet been fully reported, but published analysis so far has shown a prevalence of UdH of 29.2%, of migraine 26.7% and of TTH 12.9% [12]. UdH and migraine were therefore slightly more common in Turkey, but of similar proportions in relation to each other, while TTH in Turkey had only half the prevalence of TTH in Lithuania.

The study in Austria had different sampling methodology and included ages 10–18 years only, but used the same questionnaire [13]. This found a prevalence of UdH of 26.1%, of migraine 24.2%, of TTH 21.6% and of headache on ≥15 days/month of 3.0% – all similar to our findings in adolescents in Lithuania, except again, perhaps, for TTH. Speculation on the reason for the differences in TTH would probably be idle until more studies have been completed and ranges established in these age groups for the prevalence of each headache type.

The crucial finding here is that this study in Lithuania confirms the importance – postulated in the earlier studies [12, 13] – of including UdH in prevalence (and burden) estimates in these age groups. In our study it accounted for one third of all reported headache, as it did in Austria [13] (in Turkey the proportion was about 40% [12]). UdH is believed to represent immature forms of migraine and TTH [12], or, more correctly perhaps, migraine or TTH expressed by an immature brain, so that the characteristic features of these headaches have yet to develop. Short duration (< 1 h) is one manifestation of this. According to this belief, UdH should become less common with aging from childhood into adolescence, while migraine and TTH increase in prevalence as UdH evolves into one or the other. UdH did, indeed, reduce in this study from 38.0% of all reported headache in children to 27.4% in adolescents. The complex relationship seen between UdH and age can be explained by the interaction of these two factors: on the one hand, an overall increase in headache prevalence with increasing age; on the other, increasing headache differentiation, so that UdH declined with age as a proportion of all headache.

We have data available on adult headache in Lithuania, obtained through the Eurolight study [20], also a Global Campaign project [21]. It was conducted by visiting homes, and was smaller (*N* = 573), but used very similar diagnostic questions from the adult version of HARDSHIP [18, 21]. Gender-adjusted 1-year prevalence of migraine was 18.8% [15.9–22.3%] and of TTH 42.2% [38.3–46.3%], the former being lower and the latter higher than we saw in children and adolescents. Comparisons should be circumspect, because the adult study was limited in geographical scope (to Kaunas city and surrounding Kaunas region [21]). However, Lithuania is a small and rather homogeneous country. The bigger problem with the adult study was a non-participation proportion of 49.6% [21], very likely to introduce bias. Our interest is raised, nonetheless, by the prevalences in the adult study of pMOH (3.2% [2.0–4.9%]) and other headache on ≥15 days/month (5.4% [3.8–7.6%]), both high, but both very feasible in this former USSR country because they are similar to estimates in Russia from another Global Campaign study with the same methodology [22]. The concern generated is that we see, in our study, rapidly escalating prevalences of these disorders in adolescents. Whatever causes there may be for highly frequent headache, there is a suspicion that medication overuse is, at least in part, a behaviour learned by children and adolescents from their parents.

We need to comment on the over-reporting of HY by those with migraine (especially), TTH and UdH. The reported headache frequencies over the enquiry period of 28 days were perfectly feasible for all headache types; where there is conflict between these and reports of HY – essentially, for the episodic headaches – the former seem more probably correct despite that recall error should be greatly diminished in the latter. In adult studies, it should be noted, frequencies recalled over enquiry periods of 90 days have rather closely matched the reporting of HY, at least for all headache [23–26]. There are no published data on HY in younger age groups for comparison. We offer two explanations, neither very convincing but each, perhaps, a factor. First, children in particular tend to believe there are “right” and “wrong” answers (especially with yes/no questions), and may be influenced accordingly, more so perhaps in a school setting where they are used to taking tests. Second is sympathy-seeking by children who have just been describing their headaches.

The strengths of this study lie in its tested and validated methodology [11, 12, 15] and adequate sample size. Mandatory institutional schooling ensured the validity of schools-based sampling [14]: the exclusion of, at most, 10% of 17-year-olds would have introduced negligible if any bias. But the non-participation proportion of almost one third (32.6%) was a significant study limitation. It is clear that this introduced a gender-bias (non-participation was higher among males), but statistical correction could deal with this. We could not know whether there were other resulting biases, or, if there were, what influence they might have had. Parental consent was not forthcoming from almost one quarter (24.2%) of pupils – the primary cause of this problem. This will always be an obstacle to research in these age groups while prior written parental consent is required. It is not, generally, that parents object: they simply fail to register agreement. In non-invasive research of negligible risk to the individual and performed with the objective of benefiting large groups, ethics committees might consider giving parents opportunity to object and, in default, allowing the research with the participants’ own consent.

## Conclusions

Headache has, again, been shown to be common in children and adolescents, and undifferentiated headache has been confirmed as a headache type that must be recognised and included in accounts of headache in these age groups. These findings are of importance to Lithuania. They also contribute to knowledge and understanding of child and adolescent headache globally.

## Data Availability

The data are held on file at University of Mersin. Once analysis and publications are completed, they will be freely available for non-commercial purposes to any person requesting access in accordance with the general policy of the Global Campaign against Headache.
